# Control of blood glucose induced by meals for type-1 diabetics using an adaptive backstepping algorithm

**DOI:** 10.1038/s41598-022-16535-2

**Published:** 2022-07-18

**Authors:** Rasoul Zahedifar, Ali Keymasi Khalaji

**Affiliations:** Department of Mechanical Engineering, Faculty of Engineering, Kharazmi University, Tehran, P.O.B. 15719-14911, Iran

**Keywords:** Biomedical engineering, Mechanical engineering

## Abstract

In this study, an adaptive backstepping method is proposed to regulate the blood glucose induced by meals for type-1 diabetic patients. The backstepping controller is used to control the blood glucose level and an adaptive algorithm is utilized to compensate for the blood glucose induced by meals. Moreover, the effectiveness of the proposed method is evaluated by comparing results in two different case studies: in the presence of actuator faults and the loss of control input for a short while during treatment. Effects of unannounced meals three times a day are investigated for a nominal patient in every case. It is argued that adaptive backstepping is the preferred control method in either case. The Lyapunov theory is used to prove the stability of the proposed method. Obtained results, indicated that the adaptive backstepping controller is stable, and the desired level of glucose concentration is being tracked efficiently.

## Introduction

Diabetes mellitus is a group of metabolic diseases that leads to hyperglycemia^1^ or hypoglycemia^2^, where due to the defects in insulin secretion, insulin action, or both^3^, glucose level goes higher or lower than the safe zone, respectively^4^. According to WHO, diabetes is one of the leading causes of death in the world, while 422 million people worldwide have diabetes.

**Figure 1:**
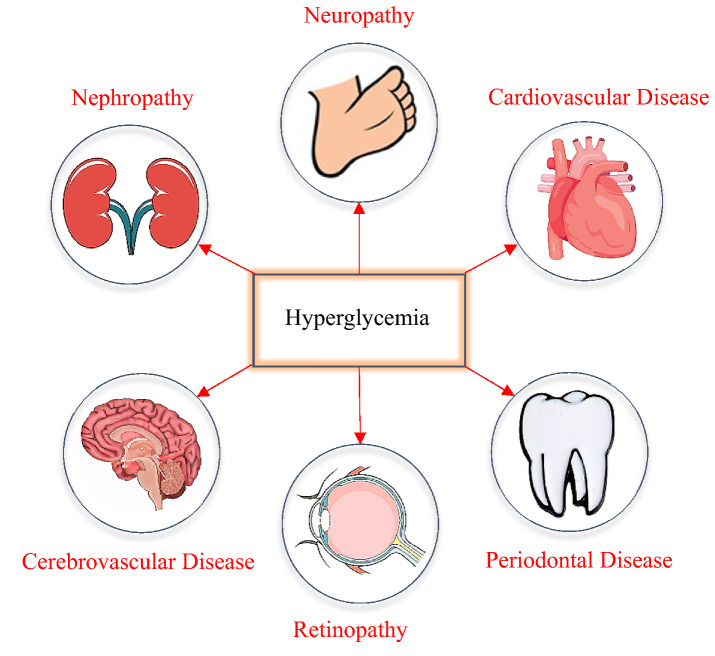
A schematic of the consequences of diabetes in the long term.

According to the American Diabetes Association, there are four types of diabetes entailing: type 1, type 2, gestational diabetes (diabetes while pregnancy), and specific types of diabetes (such as genetic defects in insulin action)^5^. Type-1 diabetes (T1D) is a chronic condition in which pancreatic $\upbeta$-cell destruction typically culminates in absolute insulin deficiency (pancreas releases little or no amount of insulin)^6^. The main symptoms of T1D are polyuria (excessive urine production), polydipsia (feeling of extreme thirstiness), and weight loss^7^. In the United States, according to CDC (Centers for Disease Control and Prevention), more than 34 million (about 1 in 10) have diabetes, where 5–10 percent have type 1 diabetes. A schematic of the consequences of diabetes in the long term is illustrated in Fig. 1. The risk of T1D is rising worldwide and nearly 90,000 children are diagnosed each year^8^. As a result, the injection of exogenous insulin, for the rest of the patient’s life, is needed to keep the glucose level of type 1 diabetes safe^9^.

Currently, no one knows how to prevent type 1 diabetes, yet we do know how to control it. The most common way is to inject insulin daily up to 4 or 5 times. Another method is the infusion of subcutaneous insulin continuously. The efficacy comparison between these two methods can be found in^10,11^. But another new promising approach was investigated by the introduction of artificial pancreas, where diabetes meets control theory. The artificial pancreas, also known as the closed-loop control of blood glucose, is a system combining a sensor, a control algorithm, and an insulin pump^12^. In this approach, the goal is to mimic the function of pancreatic insulin, in which, the sensor provides the measurements of the blood glucose concentration (BGC) and passes the information to a feedback-control system that would decide on how much insulin is needed to keep the patient’s glucose within the safe zone^13^.

To design such an artificial pancreas, several control methods and algorithms have been proposed in the literature. To name a few, a PID-based controller is proposed to provide a real-time adjustment of parameters^14,15^. In^16^, the PID controller is designed such that it is turned on only after meals and remains off before. Model predictive controller (MPC) is among the widely investigated methods^17–19^ according to its advantage; its capability to adapt itself to the changes occurring in interpatient variability as time passes. However, the efficiency of MPC depends on how much accurate the assumed model is. Another method applied in literature is fuzzy logic algorithms that require a set of rules based on advanced knowledge of the system or problem^20,21^. An adaptive control scheme is proposed in^22^, in which the controller is adjusted according to the changes in the system’s behavior. The backstepping method, firstly introduced in^23^ for nonlinear dynamical systems, is among the popular controller methods. It has a recursive design procedure and proved to be highly applicable to control blood glucose^24,25^, yet flexible to be used along with other methods, especially with adaptive control^26,27^. To bring adaptive control into the picture, the Lyapunov theory^28–30^, is the key to determining the adaptive rule. But, to control the blood glucose of T1D using the backstepping algorithm, there is still a gap in the literature that if it is advantageable to apply the adaptive control as well, to compensate for the uncertain effect of meals. There are various approaches to deal with the uncertainties of the dynamics of the system. To name a few, one technique is to use a neural network^31^, while the other one is adaptive control or a combination of both^32^. Compared to backstepping, adaptive backstepping can afford uncertainties of the model, while it might get out of control using the backstepping method. Therefore, adaptive backstepping is more reliable, especially in the presence of uncertainties, which can be seen in real-world applications. To the best of our knowledge, there is no investigation on a comparison between the efficiency of backstepping and adaptive backstepping methods to control T1D with an uncertain disturbance of meals. Furthermore, our proposed adaptive backstepping algorithm is robust in the presence of actuator faults and loss of control input for a short time, compared with the previous research on this subject in the literature.

In this paper, based on the Bergman minimal model^33^, two protocols are proposed such that blood glucose concentration tracks exponentially desired trajectories; one is achieved from backstepping and the other from adaptive backstepping. The effect of the meals, three times a day, has been considered in our analysis. Then, we claim a comparison of which method has the priority to have a better performance to control the blood glucose level of type 1 diabetic patients. Furthermore, to bring more strength to our argument, the performance of backstepping and adaptive backstepping methods are analyzed in two different case studies; in the first case study, the controllers are examined in the presence of actuator faults. In the second one, the controllers are analyzed to whether they hold their normal performance even if they confront an extremely low amount of gain affecting the input for a short while during treatment. It is concluded that under every circumstance, adaptive backstepping has the advantage.

The rest of this paper is organized as follows: the widely-used Bergman minimal model is introduced in “Mathematical model of type-1 diabetes”. Next, the desired function of glucose concentration is defined in “Control algorithm”, after which the analyses of backstepping and adaptive backstepping to achieve the final protocols are presented in “Backstepping method” and “Adapting backstepping method” respectively. This is followed by our investigation into two different case studies in “Numerical simulation”. In the end, numerical evaluation with the emphasis on comparison of the aforementioned methods, as well as case studies, are given in “Case study 1: actuator faults” and "Case study 2: controller failure for a short while".

## Mathematical model of type-1 diabetes

The dynamics model of the blood glucose-insulin system is generally non-linear. A review study about different dynamical models can be found in^34^. The most commonly used mathematical model for the blood glucose-insulin system known as Bergman minimal model was introduced in 1980^33^. In comparison with other models, the main advantage of the Bergman minimal model is its simplicity, where the relation of input and output is regulated with the minimum possible parameters, without further involvement of biological complexity. The dynamic equations of the system are as follows^35–38^:

$$\dot{G}\left(t\right)={-p}_{1}\left(G\left(t\right)-{G}_{b}\right)-G\left(t\right)X\left(t\right)+D\left(t\right)$$

$$\dot{X}\left(t\right)=-{p}_{2}X\left(t\right)+{p}_{3}\left(I\left(t\right)-{I}_{b}\right)$$

1 $$\dot{I}\left(t\right)=-n\left(I\left(t\right)-{I}_{b}\right)+u\left(t\right)$$

where $G(t)$ is the glucose concentration in the blood plasma in $\mathrm{mg}/\mathrm{dl}$, $X(t)$ is the interstitial insulin in $1/\mathrm{min}$ and $I(t)$ is the insulin concentration in the blood plasma in $\mathrm{\mu U}/\mathrm{ml}$ (or $\mathrm{\mu IU}/\mathrm{ml}$), ${G}_{b}$ and ${I}_{b}$ are the basal levels of glucose and insulin respectively, $n$ is the time constant for insulin disappearance, ${p}_{1}$, ${p}_{2}$ and ${p}_{3}$ are the insulin-independent constant rate of glucose uptake in muscles and liver, the rate for the decrease in tissue glucose uptake ability, and the insulin-dependent increase in glucose uptake ability in tissue per unit of insulin concentration above the basal level. The control input $u(t)$ in $\mathrm{\mu U}/(\mathrm{ml}/\mathrm{min})$ denotes the insulin injection rate, and $D(t)$ shows the glucose taken from meals which are uncertain in measure as a disturbance. The parameter $D(t)$ is defined by the following decaying exponential function^35^:

2 $$D\left(t\right)=A \mathrm{exp}(-Bt)$$

where $A$ and $B$ are two positive constants. The parameter values of the model (1) for a type-1 diabetic patient are represented in Table 1^13,35^.

**Table 1:** Model parameters.

Parameter	Explanation	Value	Unit
${G}_{b}$	Basal glucose level	90	mg/dl
${I}_{b}$	Basal insulin level	7	μU/ml
$n$	The time constant for insulin disappearance	0.2814	1/min
${p}_{1}$	The insulin-independent constant rate of glucose uptake	0	1/min
${p}_{2}$	The decrease rate in tissue glucose uptake ability	0.0142	1/min
${p}_{3}$	The insulin-dependent increase in glucose uptake	$1.5\times {10}^{-5}$	ml/μU/min

### Remark 1

Note that for the unit of $I\left(t\right)$, and consequently the input $u(t)$, we use $\mathrm{\mu U}/\mathrm{ml}$ (or $\mathrm{\mu IU}/\mathrm{ml}$), where $U$ ($IU$) stands for Units (International Units). However, in the International System of Units (SI), a mass-based ($\mathrm{pmol}/\mathrm{L}$) unit is used instead, yet the conversion rate is still under discussion. So, we proceed with the conventional form of the unit. For more information about the conversion rate, readers are referred to^39^.

### Remark 2

As people usually eat more at lunch, parameters $A$ and $B$ in Eq. (2) are chosen such that the lunch is taken more quantitatively than dinner and the dinner is taken more than breakfast. The values of these parameters are represented in Table 2.

**Table 2:** Disturbance parameters.

^Value^	Breakfast	Lunch	Dinner
_Parameter_			
$A$	0.4	0.6	0.5
$B$	0.01	0.01	0.01

## Control algorithm

First, a time-varying desired trajectory ${G}_{d}(t)$ is introduced as the reference signal for the glucose concentration $G\left(t\right)$ to be tracked. The signal is defined as ${G}_{d}\left(t\right)={G}_{\infty }+\left({G}_{0}-{G}_{\infty }\right)\mathrm{exp}(-t/\tau )$ so that it decreases exponentially from the initial value ${G}_{0}$ to the set final value ${G}_{\infty }=100$ with the time constant $\tau =100$ min.

Consider the error between the actual output and the reference defined as:

3 $${e}_{1}=G\left(t\right)-{G}_{d}(t)$$

From this point forward, ${x}_{1}$, ${x}_{2}$, ${x}_{3}$, and ${x}_{1d}$ are used instead of the parameters $G\left(t\right)$, $X(t)$, $I(t)$, and ${G}_{d}(t)$ respectively. Also, the notation of time (t) is removed for convenience.

### Backstepping method

In this section, the goal is to converge the error signal ${e}_{1}$ to zero exponentially. The step by step designed protocol is as follows.

#### First step

Firstly, a positive definite Lyapunov function candidate is defined as ${V}_{1}=\frac{1}{2}{e}_{1}^{2}$. If its time derivative i.e., ${\dot{V}}_{1}={e}_{1}{\dot{e}}_{1}$, is negative definite, it means ${e}_{1}$ is converging exponentially to zero. Hence the following stable error dynamics is chosen:

4 $${\dot{e}}_{1}+{\mathcalligra{k}}_{1}{e}_{1}=0$$

where ${\mathcalligra{k}}_{1}$ is a positive constant. Therefore, ${\dot{e}}_{1}$ from Eq. (4) can be applied into ${\dot{V}}_{1}$ and consequently:

5 $${\dot{V}}_{1}={e}_{1}{\dot{e}}_{1}={e}_{1}\left(-{\mathcalligra{k}}_{1}{e}_{1}\right)=-{\mathcalligra{k}}_{1}{e}_{1}^{2}\le 0$$

It can be concluded that ${e}_{1}$ is converging exponentially to zero. Also, Eq. (4), can be written as:

6 $$\left({\dot{x}}_{1}-{\dot{x}}_{1d}\right)+{\mathcalligra{k}}_{1}({x}_{1}-{x}_{1d})=0$$

Now, ${\dot{x}}_{1}$ can be replaced from Eq. (1) into Eq. (6):

7 $$\left\{-{p}_{1}\left({x}_{1}-{G}_{b}\right)-{x}_{1}{x}_{2}+D-{\dot{x}}_{1d}\right\}+{\mathcalligra{k}}_{1}\left({x}_{1}-{x}_{1d}\right)=0$$

The ${x}_{2}$ obtained from the above equation is the desired ${x}_{2}$ for the next step and it is denoted with ${x}_{2d}$. Therefore, we have:

8 $${x}_{2d}=\frac{1}{{x}_{1}}[{-p}_{1}\left({x}_{1}-{G}_{b}\right)-{\dot{x}}_{1d}+{\mathcalligra{k}}_{1}\left({x}_{1}-{x}_{1d}\right)]$$

Note that as $D$ is unknown, we are not allowed to bring it to the controller.

#### Second step

In the next step, the error signal for the actual value of the second state and its desired value is defined as ${e}_{2}={x}_{2}-{x}_{2d}$. Accordingly, the second Lyapunov function candidate is defined as ${V}_{2}=\frac{1}{2}{e}_{2}^{2}$. The same scenario for achieving ${x}_{2d}$ is applied to obtain ${x}_{3d}$. First, the desired error dynamics is selected as follows:

9 $${\dot{e}}_{2}+{\mathcalligra{k}}_{2}{e}_{2}=0$$

where ${\mathcalligra{k}}_{2}$ is a positive constant. Based on Eq. (9) we have ${\dot{e}}_{2}=-{\mathcalligra{k}}_{2}{e}_{2}$, and substituting it in the derivative of ${V}_{2}$, leads to:

10 $${\dot{V}}_{2}={e}_{2}{\dot{e}}_{2}={e}_{2}\left(-{\mathcalligra{k}}_{2}{e}_{2}\right)=-{\mathcalligra{k}}_{2}{e}_{2}^{2}\le 0$$

Therefore, the derivative of the Lyapunov function candidate ${V}_{2}$ is obtained as a negative definite function. Consequently, ${e}_{2}$ would be converging to zero exponentially. Equation (9) can be written as follows:

11 $$\left({\dot{x}}_{2}-{\dot{x}}_{2d}\right)+{\mathcalligra{k}}_{2}({x}_{2}-{x}_{2d})=0$$

Substituting the corresponding value of ${\dot{x}}_{2}$ from Eq. (1) into Eq. (11), yields:

12 $$\left\{-{p}_{2}{x}_{2}+{p}_{3}\left({x}_{3}-{I}_{b}\right)-{\dot{x}}_{2d}\right\}+{\mathcalligra{k}}_{2}\left({x}_{2}-{x}_{2d}\right)=0$$

And now, ${x}_{3}$ obtained from Eq. (12) is the desired one:

13 $${x}_{3d}={I}_{b}+\frac{1}{{p}_{3}}[{p}_{2}{x}_{2}+{\dot{x}}_{2d}-{\mathcalligra{k}}_{2}({x}_{2}-{x}_{2d})]$$

#### Third step

In the last step, the error signal ${e}_{3}={x}_{3}-{x}_{3d}$ can be calculated and its Lyapunov function candidate is chosen as ${V}_{3}=\frac{1}{2}{e}_{3}^{2}$ accordingly. Similar to the previous steps, assuming the following stable error dynamics for ${e}_{3}$:

14 $${\dot{e}}_{3}+{\mathcalligra{k}}_{3}{e}_{3}=0$$

where ${\mathcalligra{k}}_{3}$ is a positive constant. This error dynamics leads to the following negative definite function for ${\dot{V}}_{3}$:

15 $${\dot{V}}_{3}={e}_{3}{\dot{e}}_{3}={e}_{3}\left(-{\mathcalligra{k}}_{3}{e}_{3}\right)=-{\mathcalligra{k}}_{3}{e}_{3}^{2}\le 0$$

Therefore, the exponential convergence of ${e}_{3}$ to zero can be concluded. To proceed towards this goal, Eq. (14) can be written as:

16 $$\left({\dot{x}}_{3}-{\dot{x}}_{3d}\right)+{\mathcalligra{k}}_{3}({x}_{3}-{x}_{3d})=0$$

Substituting the corresponding value for ${\dot{x}}_{3}$ from Eq. (1) into Eq. (16), yields:

17 $$\left\{-n\left({x}_{3}-{I}_{b}\right)+\mathcalligra{u}-{\dot{x}}_{3d}\right\}+{\mathcalligra{k}}_{3}\left({x}_{3}-{x}_{3d}\right)=0$$

where $\mathcalligra{u}$ is the input. Therefore, the input $\mathcalligra{u}$ can be achieved from Eq. (17) as:

18 $$\mathcalligra{u}=n\left({x}_{3}-{I}_{b}\right)+{\dot{x}}_{3d}-{\mathcalligra{k}}_{3}({x}_{3}-{x}_{3d})$$

By selecting positive gains for ${\mathcalligra{k}}_{i} (i:1\stackrel{ }{\to }3)$, the control input obtained in Eq. (18) can lead ${e}_{1}$ to converge to zero exponentially, as a result, ${x}_{1}\to {x}_{1d}.$

### Adaptive backstepping method

In this section, an adaptive rule is designed to compensate for the disturbances of glucose taken from meals. A step-by-step procedure can be used until the desired input is acquired.

#### First step

In the first step, the Lyapunov function candidate is chosen as ${V}_{1}=\frac{1}{2}{e}_{1}^{2}$, which its derivative can be obtained as:

19 $${\dot{V}}_{1}={e}_{1}{\dot{e}}_{1}={e}_{1}\left({\dot{x}}_{1}-{\dot{x}}_{1d}\right)$$

Applying the corresponding value of ${\dot{x}}_{1}$ from Eq. (1) into Eq. (19) yields:

20 $${\dot{V}}_{1}={e}_{1}\left\{{-p}_{1}\left({x}_{1}-{G}_{b}\right)-{x}_{1}{x}_{2}+D-{\dot{x}}_{1d}\right\}$$

Therefore, the desired value for ${x}_{2}$ in Eq. (20) can be chosen as:

21 $${x}_{2d}=\frac{1}{{x}_{1}}{[-{\dot{x}}_{1d}+{k}_{1}{e}_{1}-p}_{1}\left({x}_{1}-{G}_{b}\right)+\widehat{D}]$$

where $\widehat{D}$ is the estimation of $D$, and ${k}_{1}$ is a positive constant. The error between $D$ and its estimation value is as $\widetilde{D}=D-\widehat{D}$. Substitution from Eq. (21) into Eq. (20), yields:

22 $${\dot{V}}_{1}=-{k}_{1}{e}_{1}^{2}-\widetilde{D}{e}_{1}$$

where the term $-\widetilde{D}{e}_{1}$ will be canceled in the next step.

#### Second step

In this step, the next Lyapunov function candidate is chosen as:

23 $${V}_{2}={V}_{1}+\frac{1}{2}{e}_{2}^{2}+\frac{1}{2\delta }{\widetilde{D}}^{2}$$

The time derivative of Eq. (23) can be written as:

24 $${\dot{V}}_{2}={\dot{V}}_{1}+{e}_{2}{\dot{e}}_{2}+\frac{1}{\delta }\widetilde{D}\dot{\widehat{D}}=-{k}_{1}{e}_{1}^{2}-\widetilde{D}{e}_{1}+{e}_{2}\left({\dot{x}}_{2}-{\dot{x}}_{2d}\right)+\frac{1}{\delta }\widetilde{D}\dot{\widehat{D}}$$

The corresponding value of ${\dot{x}}_{2}$ can be replaced from Eq. (1) into Eq. (24) and it yields to:

25 $${\dot{V}}_{2}=-{k}_{1}{e}_{1}^{2}+\widetilde{D}\left(-{e}_{1}+\frac{1}{\delta }\dot{\widehat{D}}\right)+{e}_{2}(-{p}_{2}{x}_{2}+{p}_{3}\left({x}_{3}-{I}_{b}\right)-{\dot{x}}_{2d})$$

Now, the desired value of ${x}_{3}$ is chosen as:

26 $${x}_{3d}={I}_{b}+\frac{1}{{p}_{3}}[{p}_{2}{x}_{2}+{\dot{x}}_{2d}-{k}_{2}{e}_{2}]$$

Also, the following disturbance estimation equation is considered as an adaptive rule.

27 $$\dot{\widehat{D}}=\delta {e}_{1}$$

Thus, substituting from Eq. (26) and Eq. (27) into Eq. (25), we get:

28 $${\dot{V}}_{2}=-{k}_{1}{e}_{1}^{2}-{k}_{2}{e}_{2}^{2}$$

where the derivative of ${V}_{2}$ is negative semi-definite in the next step the error signal ${e}_{3}$ is brought into the picture.

#### Third step

In the last step, ${V}_{3}$ is defined as ${V}_{3}={V}_{2}+\frac{1}{2}{e}_{3}^{2}$, which its time derivative is obtained as:

29 $${\dot{V}}_{3}={\dot{V}}_{2}+{e}_{3}{\dot{e}}_{3}={\dot{V}}_{2}+{e}_{3}\left({\dot{x}}_{3}-{\dot{x}}_{3d}\right)=-{k}_{1}{e}_{1}^{2}-{k}_{2}{e}_{2}^{2}+{e}_{3}\left({\dot{x}}_{3}-{\dot{x}}_{3d}\right)$$

By replacing the corresponding value of ${\dot{x}}_{3}$ from Eq. (1) into Eq. (29), we have:

30 $${\dot{V}}_{3}=-{k}_{1}{e}_{1}^{2}-{k}_{2}{e}_{2}^{2}+{e}_{3}\left(-n\left(x3-{I}_{b}\right)+u-{\dot{x}}_{3d}\right)$$

Therefore, the control input $u$ can be chosen as:

31 $$u=n\left({x}_{3}-{I}_{b}\right)+{\dot{x}}_{3d}-{k}_{3}{e}_{3}$$

Consequently, substituting from Eq. (31) into Eq. (30), yields:

32 $${\dot{V}}_{3}=-{k}_{1}{e}_{1}^{2}-{k}_{2}{e}_{2}^{2}-{k}_{3}{e}_{3}^{2}$$

**Figure 2:**
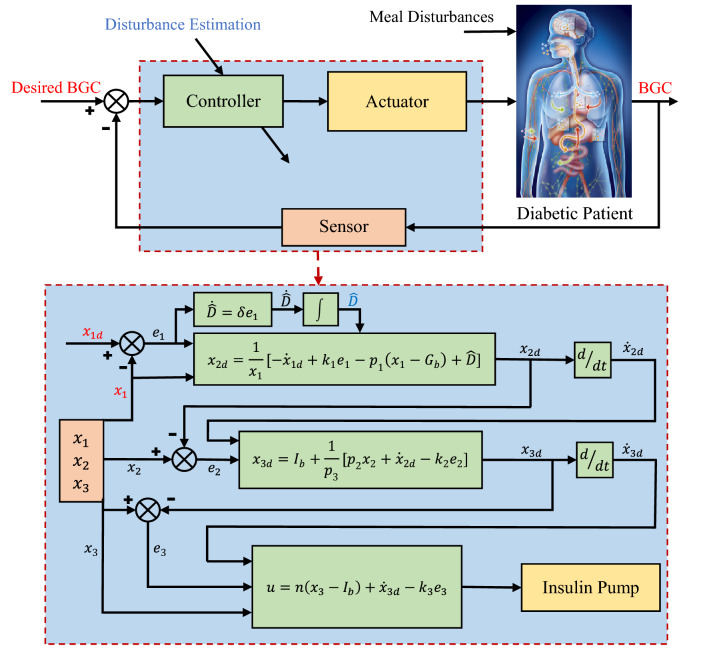
Block diagram of the adaptive backstepping algorithm proposed for the regulation of blood glucose for type-1 diabetics.

As can be seen, by choosing positive gains for ${k}_{i} (i:1\stackrel{ }{\to }3)$, ${\dot{V}}_{3}$ would be a negative semi-definite function. Regarding the reference signal ${x}_{1d}$ is an exponentially decreasing function, hence it is globally bounded, so is ${e}_{1}$. Moreover, ${\dot{x}}_{1d}$, ${x}_{1}$, and $\widehat{D}$ are also globally bounded. So, the global boundedness of ${x}_{2d}$ is concluded, and consequently, ${e}_{2}$ is globally bounded. Furthermore, ${\ddot{x}}_{1d}$, ${x}_{2}$ and $\dot{\widehat{D}}$ are also globally bounded, which yields to the global boundedness of ${x}_{3d}$ and as a result, ${e}_{3}$ is globally bounded. Hence, the function ${V}_{3}$ is globally bounded as $t\stackrel{ }{\to }\infty$ and ${\dot{V}}_{3}$ is uniformly continuous (in other words ${\ddot{V}}_{3}$ is bounded). Then by Barbalat Lemma^28^, ${\dot{V}}_{3}\stackrel{ }{\to }0$ as $t\stackrel{ }{\to }\infty$. As a result, ${e}_{1}\stackrel{ }{\to }0$ as $t\stackrel{ }{\to }\infty$, and ${x}_{1}\to {x}_{1d}$ is achieved. A schematic of how the proposed control algorithm works is demonstrated in Fig. 2, where BGC stands for blood glucose concentration. The input is insulin injection rate, while the output is blood glucose level. It should be noted using continuous glucose monitoring (CGM), the states ${x}_{1}$ and ${x}_{2}$ can be measured, while the state ${x}_{3}$ can be estimated in real time^40,41^.

## Numerical simulation

In this section, we represent numerical simulations of a type-1 diabetic patient under the Bergman minimal model and designed inputs in Eq. (18) and Eq. (31). For this purpose, we use the values of nominal parameters shown in Table 1. The simulations are investigated in a 24 h analysis, starting from fasting glucose level (no food taken for at least 8 h) at 6 A.M. The meals are taken at 8 A.M. as breakfast, 2 P.M. as lunch, and 8 P.M. as dinner. The effects of foods are placed somehow in which the lunch meal amount is more than dinner while dinner is more than breakfast. For type-1 diabetic patients, the fasting level of glucose is higher than 126 mg/dl^3^. So, we should set the initial condition ${G}_{0}$ higher than this level. The initial conditions are as follows: $G\left({t}_{0}\right)=150$ mg/dl, $X\left({t}_{0}\right)=0$ 1/min, and $I\left({t}_{0}\right)=100$ μU/ml. The gains are chosen as ${k}_{1}=0.43$, ${k}_{2}=0.46$, ${k}_{3}=0.62$, analogous for both methods, with $\delta =0.001$ as the adaptive rule gain.

**Figure 3:**
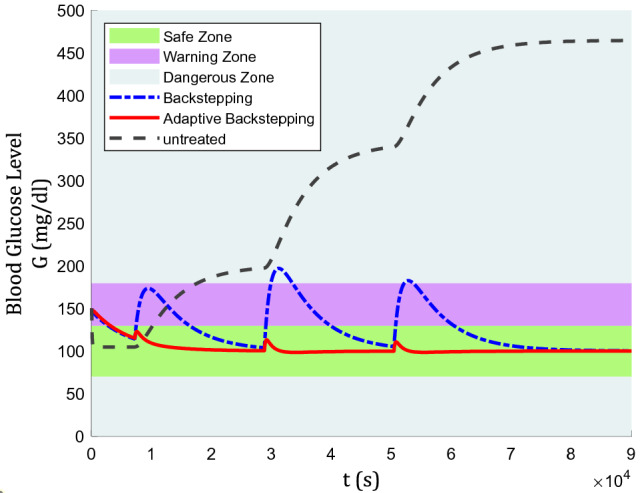
Blood glucose level for a nominal patient under the control algorithm.

The blood glucose level for a nominal patient under the control algorithm is depicted in Fig. 3. In Fig. 3, there are three colored zones divided by their safety level for type-1 diabetic patients. the zones are classified into the safe zone, warning zone, and dangerous zone. The area above 180 mg/dl (hyperglycemia) and below 70 mg/dl (hypoglycemia) are labeled as dangerous zone, between 130 mg/dl and 180 mg/dl as the warning zone, and between 70 mg/dl and 130 mg/dl is the safe zone.

It can be easily seen that without treatment, the blood glucose level rises to a dangerous level, which proves that insulin for type-1 is not required for the control, but for survival^3^. Furthermore, regarding the efficiency of backstepping and adaptive backstepping methods, backstepping has been performed mostly in warning zone, even touching dangerous zone after lunch and dinner meals are taken. While, adaptive backstepping has shown a satisfying control performance as it keeps the glucose level in the safe zone, even during mealtime. Using the adaptive backstepping technique, a lunch meal with its huge influence could only increase blood glucose from 100 mg/dl to almost 112 mg/dl.

**Figure 4:**
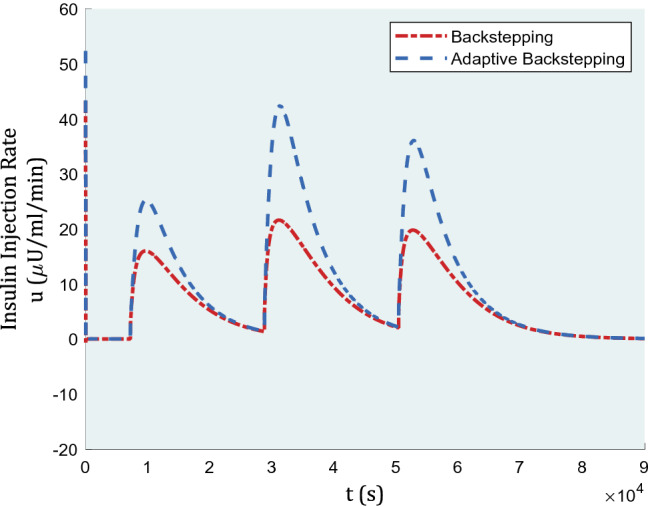
Insulin injection for a nominal patient under the control algorithm.

In Fig. 4, the graph of inputs is represented for comparison of backstepping and adaptive backstepping algorithms. In the beginning, as the fasting blood glucose rate was assumed to be matched with uncontrolled type-1 diabetes, the inputs confront jumps in insulin rate to compensate for high blood glucose levels as soon as possible. The insulin injection amounts are within reasonable ranges as almost 40 μU/ml is required during lunch for adaptive backstepping. The more insulin injected, the more decreasing blood glucose level could be, yet backstepping performance is not rewarding with a lesser insulin rate. Arguably, we do not have limits to use more insulin dosage within practicable range, especially when it is humans’ lives under discussion. Having the same controller gains, backstepping failed to apply more insulin amounts to show a better, yet necessary performance.

**Figure 5:**
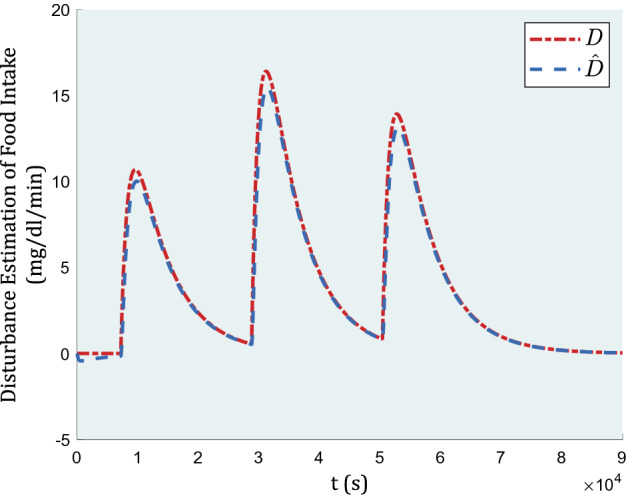
Estimation of blood glucose induced by meals as a disturbance.

In Fig. 5 the estimation of blood glucose induced by meals as a disturbance is demonstrated.

Figure 5 indicates how well-ordered the proposed disturbance estimation is comparatively following its actual value. The adaptive backstepping advantage is relied on how efficiently the adaptive rule works.

**Figure 6:**
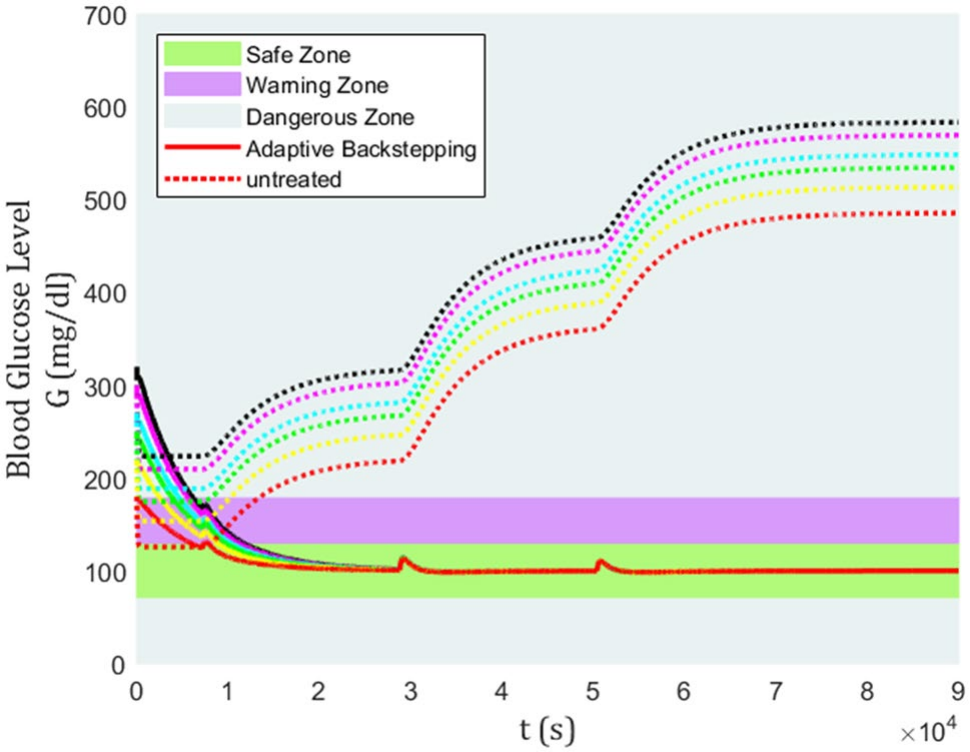
Blood glucose control with different initial conditions under the proposed control algorithm.

In the last step, the graph shown in Fig. 6 represented the effectiveness of the adaptive backstepping algorithm to control the blood glucose level of the nominal patients with different initial conditions. Starting from even the harshest initial condition, with blood glucose level at 320 mg/dl, the safe zone is gradually obtained only 75 min after breakfast is eaten.

## Case study 1: actuator faults

It is not deniable that the actuators may become obsolete after a while and show signs of faults in their performance. But the controller should be designed in advance such that it is robust towards actuator faults. In this section, the performance of backstepping and adaptive backstepping methods are compared under such conditions. To implement this purpose, multiplicative and additive actuator faults are applied to the controller in the form of:

33 $${u}_{faulty}(t)=\rho \left(t\right)u\left(t\right)+\varphi (t)$$

where $\varphi (t)$ indicates the additive actuator fault and $\rho \left(t\right)$ is the multiplicative actuator fault, such that $0<\rho (t)\le 1$. Actuator faults are applied as harshly as possible; therefore, it would be a challenging task for the proposed algorithm. Towards this goal, the parameters are set as follows: $\rho \left(t\right)=0.01+0.99\mathrm{exp}(-0.1t)$ and $\varphi \left(t\right)=0.1(1-\mathrm{exp}(-0.1t))$.

### Remark 3

Although it is not very realistic to design the actuator fault this much more severe, the faultier it is, the more robust the proposed algorithm can be claimed.

### Remark 4

The additive fault, evident from its name, is a kind of fault added to the channel of control input separately. While, multiplicative fault steps on the normal value of input as a time-dependent gain, the more $\rho (t)$ closed to zero, the faultier, and consequently weaker, the input becomes^42^.

**Figure 7:**
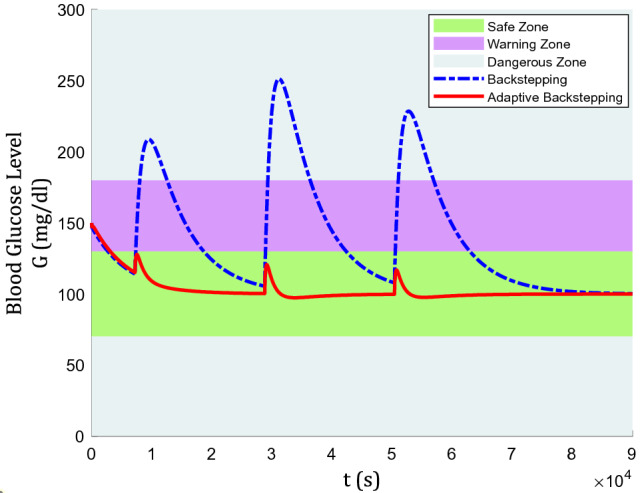
Blood glucose level in the presence of actuator faults under the control algorithm.

The blood glucose level in the presence of actuator faults under the control algorithm is demonstrated in Fig. 7.

In Fig. 7, it is again obvious that the adaptive backstepping algorithm can control blood glucose even under such harsh conditions of actuator faults. However, the backstepping algorithm failed as the blood glucose level surged towards almost 250 mg/dl, while it was around 200 mg/dl without actuator faults after lunch was taken. On the contrary, for adaptive backstepping, the glucose level peaks at 120 mg/dl and 113 mg/dl, with and without actuator faults, respectively.

**Figure 8:**
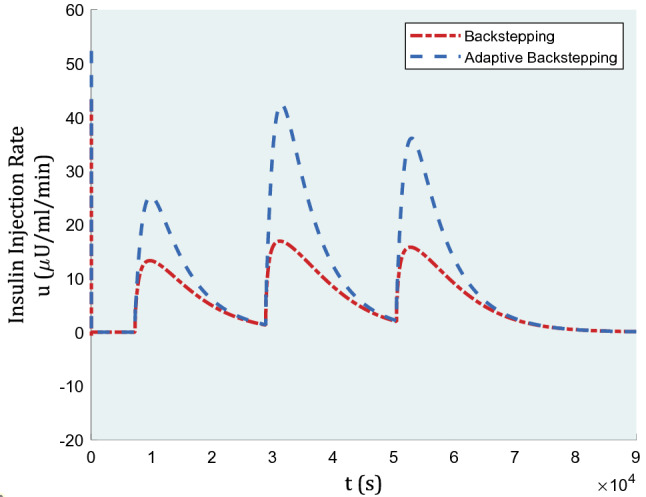
Insulin injection in the presence of actuator faults under the control algorithm.

Insulin injection in the presence of actuator faults under the control algorithm is displayed in Fig. 8.

**Figure 9:**
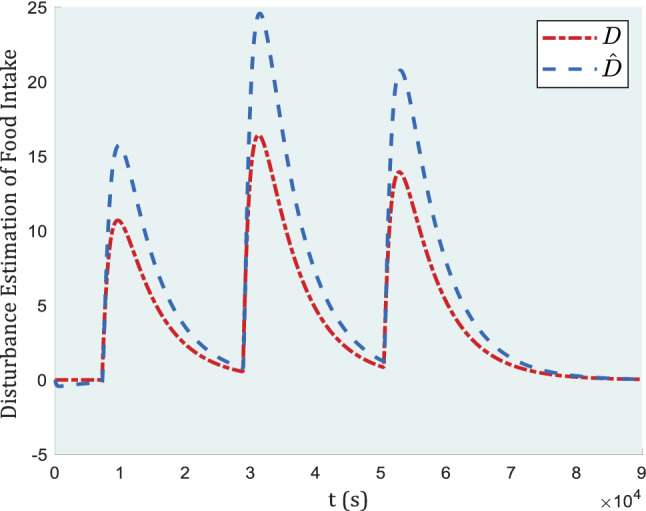
Estimation of blood glucose induced by meals as a disturbance in the presence of actuator faults.

In Fig. 8, the graph of control inputs is given to indicate that the value of the input has remained in a reasonable range. Even the gains of the system to keep the adaptive backstepping algorithm well-performed, are still the same as without actuator faults. The difference can be seen in Fig. 9, as the disturbance estimation transcends its actual value and still can keep adaptive backstepping working properly.

In Fig. 9, compared to Fig. 5, where the disturbance estimation tracked its true value almost accurately, the parameter $\widehat{D}$overestimated parameter D, especially around meal time. This is due to the existence of actuator fault, which are considered to be more severe and far from reality to evaluate the robustness of the controller. The faultier the actuator, the more robust the proposed algorithm can be claimed.

While the disturbance is overestimated due to an actuator fault, the controller tries to correct the faulty input effect by estimating the disturbance. As shown in Fig. 7, blood glucose levels return to the safe zone, but despite the high fault of the actuator, ideal answers can not be expected.

## Case study 2: controller failure for a short while

As discussed before, insulin is required for the survival of type-1 diabetic patients. But what happens if the controller almost fails to work for a short while. The algorithm should be examined to the extent that if such a condition happens, it would not culminate in disaster for patients. To investigate this case study, control inputs are designed as follows:

34 $$u=\left\{\begin{array}{cc}u & t<10\mathrm{ A}.\mathrm{ M}.\mathrm{ or }t>12\mathrm{ P}.\mathrm{M}.\\ 0.002u& 10\mathrm{ A}.\mathrm{ M}. <t<12\mathrm{ P}.\mathrm{M}.\end{array}\right.$$

where, between 10 A.M. and 12 P. M., a very low amount of gain is multiplied by the input value. The efficiency of the adaptive backstepping algorithm is concluded one more time, compared to backstepping, to control blood glucose concentration.

**Figure 10:**
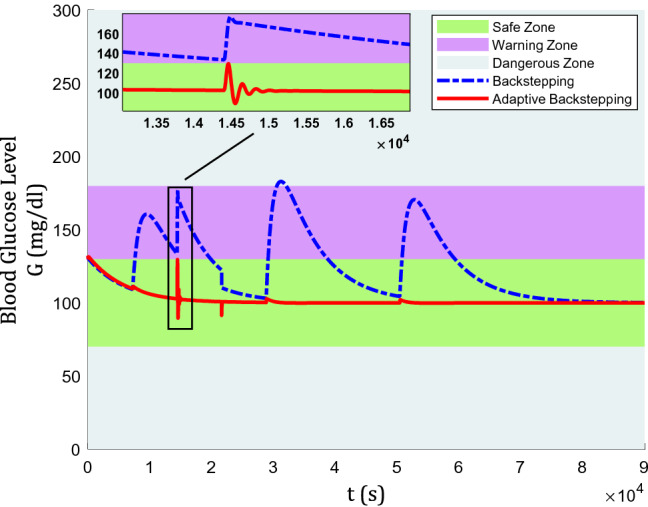
Blood glucose level in the 2-h absence of controller.

In Fig. 10, the graph of blood glucose levels is presented where adaptive backstepping still holds the advantageable place. Noticeably, the appropriate reaction of adaptive backstepping to this condition is gentler while quicker. Adaptive backstepping jumps from almost 100 mg/dl to 130 mg/dl and comes back to its normal trend in only 15 min. However, backstepping increases from almost 130 mg/dl to 175 mg/dl, and it takes more than 1 h to get back to its previous state. The considerable fact is that, during this process, adaptive backstepping remains in the safe zone, while backstepping takes steps nearer to the dangerous zone.

**Figure 11:**
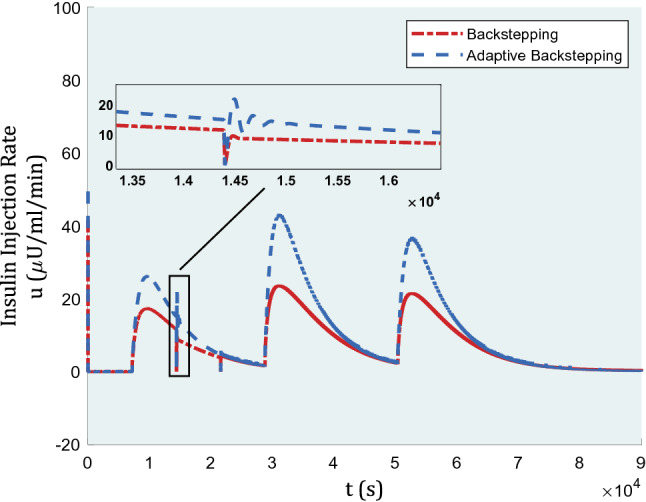
Insulin injection in the 2-h absence of controller.

**Figure 12:**
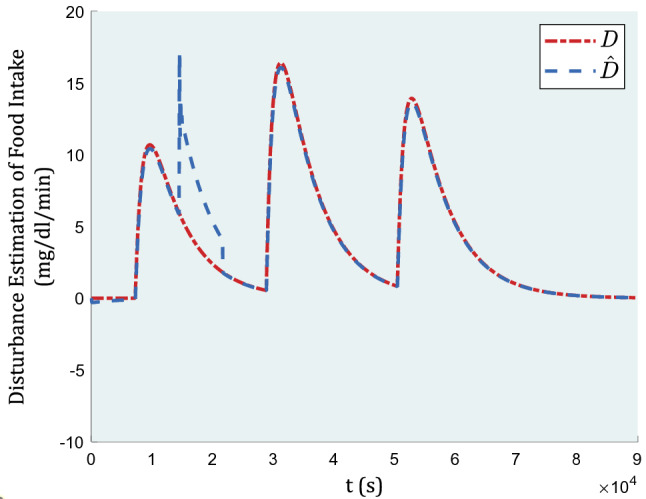
Disturbance estimation in the 2-h absence of controller.

In Figs. 11 and 12, the graph of inputs and disturbance estimation under this case study are depicted, respectively.

In Fig. 11, a small amount of deviation is seen at the start of this 2-h-period. The range of inputs almost stands the same as the former ones, though the gains are not alike. The gains are ${k}_{1}=0.45$, ${k}_{2}=0.45$, and ${k}_{3}=1.5$ similar for both methods, with $\delta =0.007$ as the adaptive rule gain.

## Conclusion

Based on the Bergman Minimal model of glucose-insulin level of type-1 diabetics, the adaptive backstepping method had been proposed and compared with the backstepping algorithm. The effects of the meal taken three times a day had been considered in the model. The effectiveness of the adaptive backstepping method had excelled over that of the backstepping algorithm. Moreover, to indicate that adaptive backstepping is more robust in different conditions compared to backstepping, two case studies were investigated. One in the presence of the actuator faults and the other in the presence of an extremely low amount of gain to act on input for a short while. The efficiency of the proposed algorithm had been analyzed using numerical comparison results. All situations confirmed that adaptive backstepping had been much more promising than the backstepping method to control the blood glucose level of type-1 diabetic patients.

## Data Availability

No datasets were generated or analysed during the current study.
